# Orthogeriatric Assessment of the Elderly Patient with Fragility Hip Fracture: Preliminary Results of a Prospective Study

**DOI:** 10.3390/jpm13071138

**Published:** 2023-07-14

**Authors:** Francesco Salis, Benedetta Puxeddu, Veronica Piras, Maristella Belfiori, Giuseppe Marongiu, Antonio Capone, Antonella Mandas

**Affiliations:** 1 Department of Medical Sciences, and Public Health, University of Cagliari, 09100 Cagliari, Italy; 2 Department of Medicine, Surgery, and Pharmacy, University of Sassari, 07100 Sassari, Italy; 3 University Hospital “Azienda Ospedaliero-Universitaria” of Cagliari, 09042 Monserrato, Italy; 4 Department of Surgical Sciences, University of Cagliari, 09100 Cagliari, Italy

**Keywords:** comprehensive geriatric assessment (CGA), hip fracture, fragility fracture, elderly, frailty, orthogeriatrics

## Abstract

Nowadays, more studies deal with “OrthoGeriatrics”, for the co-management of elderly patients suffering fractures, from the admission to the discharge and beyond. For the first time at Cagliari University Hospital, we introduced an orthogeriatric service, in which trained geriatricians stay in orthopedic unit alongside trained orthopedics. The primary aim of the study was to analyze the rate of death and rehospitalization in elderly femur-fractured people of 65 or more years of age, identifying possible predictive factors. The secondary aim of the study was to analyze the recovery of daily living autonomies during the months following surgery. To reach the aim, we designed a prospective study, which is currently ongoing. We evaluated femur-fractured patients aged 65 years or more with a comprehensive geriatric assessment before surgery. The most common fractures were lateral hip ones, treated with osteosynthesis. Cognitive–affective, functional, and nutritional status, mood, and comorbidities were less impaired than in the outpatient service of the same hospital devoted to frail elderly. Pain control was excellent. A significantly low delirium incidence was found. More than a third of the sample were recognized as frail (according to the Survey of Health, Ageing and Retirement in Europe—Frailty Instrument (SHARE-FI)), and over a third of the sample were identified as a moderate-high risk of hospitalization and death (according to Multidimensional Prognostic Index (MPI)). Overall mortality rate was 13.87%, and rehospitalization rate was 11.84%. Frail people were more likely to die than non-frail (HR: 5.64), and pre-frail ones (HR: 3.97); similarly, high-risk patients were more likely to die than low-risk (HR: 8.04), and moderate-risk ones (HR: 5.46). Conversely, neither SHARE-FI nor MPI predicted rehospitalization. Creatinine (OR: 2.66, *p* = 0.003) and folate (OR: 0.75, *p* = 0.03) levels were independently associated with death and rehospitalization, respectively. Finally, the patients did recover the lost autonomies later, 6 months after surgery. Our study demonstrated that SHARE-FI and MPI are reliable tools to predict mortality in an orthogeriatric setting, and that creatinine and folate levels should also be measured given their independent association with negative outcomes.

## 1. Background

Demographic prospects foresee further life expectancy increments worldwide [1,2]. As the population grows, geriatric medicine grows as well. Such a scenario increases the likelihood of a future higher presence of age-related diseases, such as various levels of cognitive impairment [3,4], hypertension [5], and fractures [6], as well as malnutrition and polypharmacotherapy [7]. Comprehensive geriatric assessment (CGA) still represents the tool capable of holistically frame old subjects [8], since these patients usually need medical interventions in more than one domain [9]. The Multidimensional Prognostic Index (MPI) [10] is a tool assessing various geriatric domains, validated in an acute care setting, and applied in several types of patients and co-morbidities [11,12,13], which gives a mid-term adverse event risk, namely death and rehospitalization/institutionalization, dividing the patients into three categories (mild, intermediate, and severe) according to its scores. It was also related to the frailty [14], following the need to characterize it with validated and multidimensional tools rather than the less precise “phenotypes” [15]. To the best of our knowledge, the first validate tool with this purpose is the Survey of Health, Ageing and Retirement in Europe—Frailty Instrument (SHARE-FI) [16], which in turn divides patients into three categories (non-frail, pre-frail, and frail), offering different scoring for males and females. It is known that frailty is responsible for increased deaths in patients affected by different pathologies [17,18,19,20,21,22], as well as their early assessment [23,24].

The abovementioned data highlight the interest of scientific research in characterizing the need to care for more people. Among them, a particular population is represented by the fractured. According to the epidemiological data, fractures are one of the most common comorbidities in elderly [6,21], being particularly significant not only for their own health, but also from an economic and of public health point of view [18,19,20]: fractures have impact on social costs and long-term disability in such a particular population. Since mineral bone mass decreases as age increases, due to different pathophysiological reasons [25,26], the term “fragility fractures” was coined to refer to an event usually occurring in elderly people with osteoporosis due to low-energy trauma on low-quality bone [27,28]. Hip-fracture is the most common [29], with a mortality ranging from 14% to 36% considering all ages, and surgery is the best treatment considering the risk/benefit balance [30]. Among fragility fractures, it is necessary to also mention upper extremity fractures [31], especially distal radius, pelvis ring [32], and vertebral ones [33].

Since the mentioned events result in increased deaths and comorbidities, and loss of autonomy in the elderly, it would be desirable to early identify more compromised and at-risk subjects [28], and all these factors resulted in the birth of “OrthoGeriatrics” [34], namely services in which orthopedic doctors and geriatricians cooperate in order to optimize fractured patients’ management [35], from hospitalization to the discharge and beyond.

Taking everything into consideration, the primary aim of this study is to analyze the rate of death and rehospitalization in elderly proximal femur-fractured people of 65 or more years of age, weighted according to MPI and SHARE-FI, identifying possible predictive factors.

The secondary aim of this study is to analyze the recovery of daily living autonomies during the months following surgery.

## 2. Methods

### 2.1. Design of the Study

This prospective study included subjects evaluated at the Orthopedic Unit of the University Hospital of Monserrato, Cagliari, Italy, from November 2021 to November 2022, and followed-up at one, three, and six months after surgery. The study protocol foresees a 1-year follow-up, which is currently ongoing.

### 2.2. Inclusion Criteria

The inclusion criteria were as follows: age ≥ 65 years; presence of proximal femur fracture; having been subjected to CGA; having been subjected to surgery.

### 2.3. Exclusion Criteria

The exclusion criteria were as follows: age < 65 years; absence of femur fracture; not having been subjected to surgery; informed consent not provided.

### 2.4. Assessment

Within a day of admission (t0) the subjects were subjected to CGA, including the following.

Assessment of mid-term risk of adverse event (rehospitalization, death) with the Multidimensional Prognostic Index (MPI) [10], which ranges from 0 to 1, divided into MPI1 (low risk, 0.0–0.33), MPI2 (moderate risk, 0.34–0.66), and MPI3 (severe risk, 0.67–1.0). It includes the following:

Social support (household composition, institutionalization, services).

Cognitive assessment with the Short Portable Mental Status Questionnaire (SPMSQ) [36]. It ranges from 0 (absence of cognitive impairment) to 10 (maximum impairment). Scores < 5 indicate absence or mild impairment, scores from 5–7 indicate moderate impairment, and scores from 8–10 indicate severe impairment.

Residual autonomies assessment with Activities of Daily Living (BADL) [37]—ranging from 6 (complete independence) to 0 (complete dependence)—and Instrumental Activities of Daily Living (IADL) [37]—ranging from 8 (independence) to 0 (complete dependence).

Nutritional status assessment with the Mini Nutritional Assessment (MNA) [38]. It ranges from 30 (excellent nutritional status) 0 (severe malnutrition), where scores < 17 indicate malnutrition, scores 17–23.5 indicate risk of malnutrition, and ≥24 indicates adequate nutritional status.

Pressure injuries risk assessment with the Exton-Smith Scale (ESS) [39]. It ranges from 20 (absence of risk) to 5 (maximum risk), and scores ≤ 12 indicate a surely increased risk.

Comorbidities assessment with the Cumulative Illness Rating Scale (CIRS) [40], which evaluates 14 categories of pathologies, hypertension, and psychiatric and behavioral aspects. The Complex Comorbidity Index (CIRS CCI), included in MPI measurement, corresponds to the number of at least needing treatment categories.

Number of different drugs taken.

Mood assessment with the Geriatric Depression Scale (GDS) [41]. It ranges from 0 (complete absence of depression) to 15 (severe depression), and scores ≥ 5 indicate presence of depression.

Pain assessment with the Numeric Pain Rating Scale (NPRS) [42]. It ranges from 0 (pain absent) to 10 (strongest pain imaginable).

Delirium assessment with the 4 “As” Test (4-AT) [43] and the Brief Confusion Assessment Method (bCAM) [44].

Frailty assessment with the Survey of Health, Ageing and Retirement in Europe—Frailty Instrument (SHARE-FI) [16]. It evaluates five domains (exhaustion, loss of appetite, muscular strength with a dynamometer, walking capacities, physical activity), and divides patients into frail, pre-frail, and non-frail categories, and also according to gender.

After one (t1), three (t2), and six months (t3) after surgery, patients were subjected to:

Anamnesis to subsequent hospitalizations and/or death.

Residual autonomies assessment with BADL and IADL.

The abovementioned tests were administered by trained geriatricians.

### 2.5. Statistical Analysis

Variables were expressed as means and SDs or in percentages (%), where appropriate. Kaplan–Meier curves were designed in order to estimate the survival probability. The comparison of survival curves between the two groups was studied with the log-rank test, and expressed as χ² and C.I., while the differences in time of the event occurring were expressed as hazard ratios (HRs). Multivariate analysis was performed using a stepwise Cox regression (*p*-values > 0.1 were excluded by the model). The two outcomes were rehospitalization and exitus, and age, gender, length of hospitalization, before surgery status, type of surgery, cognitive, functional, and nutritional status, pressure injury risk, mood, the most common drugs taken and comorbidities, hemoglobin, renal (creatinine) and thyroid (thyroid-stimulating hormone) function, iron status (iron, ferritin), albumin, vitamin B12, folates, and vitamin D were the possible predictors. The results were expressed as odds ratios (ORs) and confidence intervals (C.I.). ANOVA for repeated measures was performed to analyze the variance among the variables during the follow-up period. The Bonferroni model was used for post hoc analysis.

The results are reported indicating *p*-values in reference to 95% C.I.

MedCalc software (Version 20.218, Ostend, Belgium) was used for the statistical analysis.

## 3. Results

**Figure 1:**
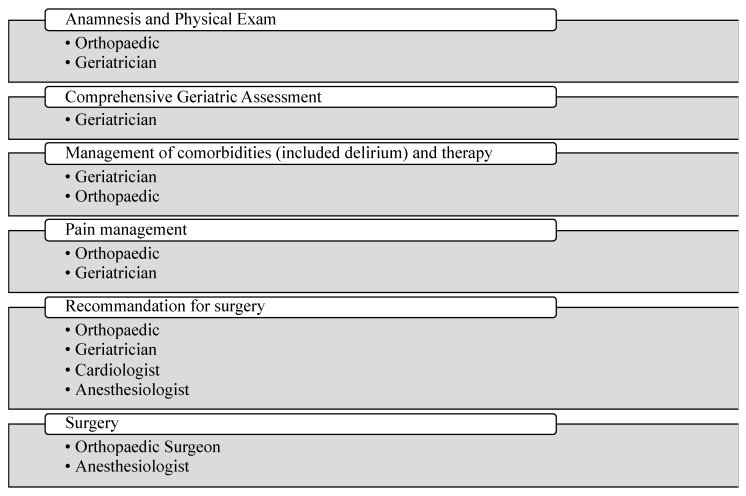
Co-management.

The study enrolled patients having been admitted to the orthopedical unit due to fractures: within the 13 months of enrollment, 333 subjects, of whom 249 (74.8%) were women, with an average age of 83.7 years, have undergone geriatric examination and CGA within a day of admission. The patients’ co-management is explained in Figure 1.

Subjects with proximal femur fracture who have been subjected to surgery, also underwent a follow-up at 1 (t1), 3 (t2), and 6 months (t3) after surgery, in order to evaluate the frequency of negative events (rehospitalization, exitus) and the remaining autonomies in basic and instrumental activities of daily living. Since the present study analyzes data referring to the period November 2021–November 2022, 68.2% completed the 1-, 3-, and 6-month follow-up period, 8.2% completed only 1- and 3-months, while 5.7% completed only 1-month. We specify that the study is still ongoing, and one-year follow-up is expected to occur as well.

The characteristics of the subjects are summarized in Table 1, Table 2 and Table 3.

The average time between admission and surgery was 6.2 days (ranging from 0 to 37), and the average length of hospitalization was 15.7 days (ranging from 0 to 62). Lateral fractures were more common, and were treated with osteosynthesis (60.9%), followed by medial fractures (39.1%), which were treated with arthroplasty.

At the moment of admission, concerning cognitive–affective evaluation, 25.3% of the sample showed cognitive impairment, and 15.9% showed a depressed mood; functional evaluation (referred to the status before the event) showed that 23.7% and 29.4% of the patients were rated 2 or more for BADL and 4 or more for IADL, respectively. An increased risk for pressure injuries was found in 20%. Concerning nutritional status, 40.8% were considered at risk of malnutrition, while 4.9% were openly malnourished. At the time of assessment (under pain therapy), 5.3% complained of moderate pain according to NPRS. Seven-point-three percent of the sample had socio-environmental problems (understood as, i.e., destitution or lack of cooperation from family members).

By considering 4-AT, 9.7% of the sample presented delirium during the hospital stay, of whom 8.9% showed it upon admission. This number lowers to 2% considering B-CAM. Four-point-five percent contracted the SARS-CoV-2 infection during the stay. In the same period, 1% of the patients died.

With regard to hematochemical tests, hemoglobin levels < 13 g/dL were found in 66.2% of the subjects, and serum iron was averagely low (33.2 mg/dL), with non-elevated ferritin (201.4 μg/L), as well as serum albumin (2.9 g/dL), and vitamin D (18.6 ng/mL); only 15.5% of the sample presented values ≥ 30 ng/mL, while 48.97% presented values < 18 ng/mL.

The most common drug classes already taken before the admission were anti-hypertensive drugs (beta-blockers (30.2%), ACE inhibitors (27.8%), sartans (24.1%), thiazide diuretics (18.8%), loop diuretics (18%), and calcium-channel antagonists (18.4%)), statins (31.4%), protonic pump inhibitors (26.1%), benzodiazepines (23.7%), and antidepressants (selective serotonin reuptake inhibitors: 11%, and serotonin–norepinephrine reuptake inhibitors: 4.9%). Each patient was taking an average of 4.3 different active components, and the condition of polypharmacotherapy affected 46.1% of the sample. Furthermore, the average CIRS was 25.6, and the average number of comorbidities was 4.9; among them, hypertension was the most frequent (70.6%), followed by anemia (66.2%) dyslipidemia (29%), and osteoporosis (15.9%). In addition, 29% of the subjects had had at least one previous bone fracture.

Frailty status was assessed with SHARE-FI: 26.5% of the sample was considered “non-frail”, 19.2% “pre-frail”, and 35.9% “frail” (in 18.4% it was not applicable, due to concomitant fractures of the upper limb, or alterations in the state of consciousness). MPI was applied in order to establish a middle-term risk of negative event (hospitalization, exitus): 57.1% showed a mild risk, 31.4% a moderate risk, and 6.9% a high risk (in 4.6% it was not applicable).

**Figure 2:**
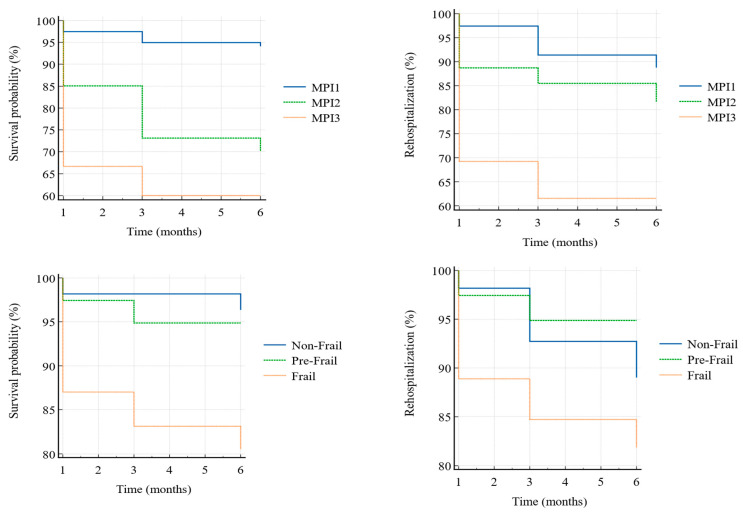
Kaplan–Meier weighted curves. MPI1, low risk; MPI2, intermediate risk; MPI3, high risk.

Following the aims of the study, we analyzed the data resulting from patients’ follow-up. The Kaplan–Meier curves show a cumulative 11.84% of rehospitalization (18.37% missing)—the most common causes were medical conditions (12 patients), followed by surgical complications (6 patients), and other causes (1 patient)—and a 13.87% of exitus (also 13.87% missing)—the most common causes of which were medical conditions (6 patients), followed by surgical complications (2 patients). The other patients’ rehospitalization and death’s causes were unspecified. We weighted such outcomes with the frailty condition and the middle-term probability of negative events (Figure 2). As in Table 3, concerning survival probability, it increased when SHARE-FI decreased (log-rank χ² = 10.05, *p* = 0.0066)—with HR = 5.64 (95% C.I. 2.00–15.91) between non-frail and frail, and HR = 3.97 (95% C.I. 1.25–12.59) between pre-frail and frail (HR between non-frail and pre-frail was non-significant). Survival probability also increased with decreasing MPI (log-rank χ² = 25.74, *p* < 0.0001)—with HR = 5.46 (95% C.I. 2.59–11.48) between MPI1 and MPI2, and HR = 8.04 (95% C.I. 1.97–32.75) between MPI1 and MPI3 (HR between MPI2 and MPI3 was non-significant). About rehospitalization, it was not related to SHARE-FI (log-rank χ² = 4.13, *p* = 0.127) nor to MPI (although log-rank χ² = 8.70, and *p* = 0.0129, none of the HRs were significant, as shown in Table 4).

Then, we conducted two Cox regressions for exploring the relationship between the two outcomes (rehospitalization and exitus), and, as covariates, we chose age, gender, length of hospitalization before surgery, type of surgery, cognitive, functional, and nutritional status, pressure injury risk, mood, the most common drugs taken, and comorbidities, as well as hemoglobin, renal (creatinine), and thyroid (thyroid-stimulating hormone) function, iron status (iron, ferritin), albumin, vitamin B12, folates, and vitamin D. According to the regression models, as in Table 5, age (OR = 1.12, *p* = 0.046), creatinine levels (OR = 2.66, *p* = 0.003), and vitamin B12 levels (OR = 1.004, *p* < 0.0001) were positively independently associated with death probability, while BADL showed a trend without reaching statistical significance (Harrell’s C-index: 0.828, *p* < 0.0001). Furthermore, as in Table 6, iron (OR = 1.03, *p* = 0.026) and vitamin B12 (OR = 1.002, *p* = 0.025) levels were positively independently associated with rehospitalization, while folate levels (OR = 0.75, *p* = 0.03) were negatively associated (Harrell’s C-index: 0.533, *p* = 0.0003).

**Figure 3:**
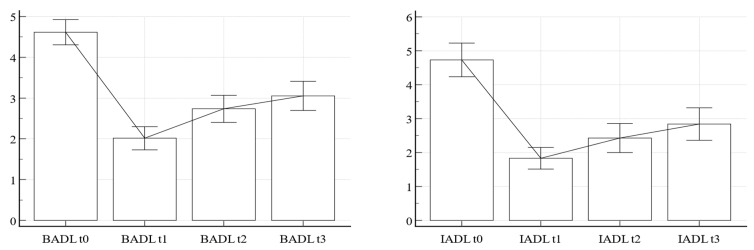
BADL and IADL follow-up. BADL, Activities of Daily Living; IADL, Instrumental Activities of Daily Living; t0, before fracture; t1, 1 month after surgery; t2, three months after surgery; t3, 6 months after surgery.

Finally, as in Figure 3, ANOVA reflects the modification of autonomies in BADL and IADL from the moment before the accident to six months after surgery, through the intermediate stages at one and three months. In BADL and IADL scores it can be seen that one month after surgery there is a linear loss of autonomy (*p* < 0.0001), with a linear recovery at t1 (*p* < 0.0001), t2 (*p* < 0.0001), and t3 (*p* = 0.0054 for BADL—mean 3.05, and *p* = 0.0033 for IADL—mean 2.84), though maintaining themselves significantly lower than before the traumatic event (*p* < 0.0001).

## 4. Discussion

Nowadays, a growing number of studies tends to deal with “OrthoGeriatrics” [34], meaning the co-management of elderly patients suffering fractures from the admission to discharge and beyond, by different health professionals (orthopedics and geriatricians among them).

For the first time at Cagliari University Hospital, in November 2021, we introduced the orthogeriatric service, in which trained geriatricians stay in the orthopedic unit in the daytime, collaborating with orthopedic doctors and other professionals, thus, avoiding the annoying practice of asking for geriatric advice when needed. In this work, we present the preliminary results of this new service, with the aim to evaluate post-surgical outcomes (loss of autonomy, disability, hospitalization, exitus), and to try to individuate factors which could predict them. We have collected data until November 2022 so far, so only 68.2% could undergo the 6-month follow-up.

With respect to the guidelines of clinical practice [46], we found that the average time between admission and surgery, where indicated, was longer. It appears to be worse than reported in other orthogeriatric services [47,48], but we believe that it could depend on a particular high age (with a maximum of 105 years) and comorbidity burden. Such an aspect represents a significant concern, since it can determine perioperative complications, and influence functional recovery and survival [49,50,51]. As such, it is necessary to consider some issues: first, the study was conducted during the COVID-pandemic, which represented an obstacle for patients to receive a ready surgical treatment; second, the number of health professionals in our unit did not allow us to carry out surgery during weekends (and this aspect surely caused the delay); third, the multivariate analysis showed that the abovementioned time was not associated with mortality nor rehospitalization, so, even if the literature reported the correlation with negative outcomes, we did not find it in our sample.

The most common fractures, according to the literature, were lateral proximal femur ones [29], for which osteosynthesis [52] was the first-line treatment. By considering geriatric assessment, namely cognitive–affective, functional, and nutritional status, mood, and comorbidities, we found slightly better findings than in an outpatient setting [9]. By the way, as is reasonably feasible, we become aware of the high number of comorbidities, among them hypertension, anemia, and previous fractures, and the spreading condition of polypharmacotherapy [53] in our sample (affecting nearly the half), which was demonstrated to be a significant issue in public health, as patients were also subjected to dangerous under- or over-prescriptions [54,55]. On the other hand, pain control was excellent and, in fact, the average NPRS values were lower than 3. These surprising data, far better than the current scientific evidence [56], are represented by the low incidence of delirium: if we consider the less specific screening tool, the 4-AT, we find less than 10% people affected by this condition; by using a second level screening tool, as the B-CAM is, the number drops to 2%. As reported, these values are significantly lower than what is reported in the scientific literature, even considering orthogeriatric settings [57,58]. We believe that these data are the results of two elements: firstly, the rapid patients’ assessment, followed by an optimal pre- and post-surgery management of pain and comorbidities; secondly, the use of validated tools to recognize delirium instead of a sole clinical evaluation, which can hardly discriminate it from other causes of psychomotor agitation. Another positive element is represented by the low number of deaths during hospitalization, once again tied to the rapid recognition and management of incipient medical diseases.

Significant interest in geriatric practice and research is given to frailty [15]. According to SHARE-FI, more than a third of the sample was recognized as frail. Moreover, the MPI recognized over a third of the sample as at a moderate–high risk of hospitalization and death. We followed-up the patients in order to demonstrate how these tools could predict actual deaths and rehospitalizations. By weighting the outcome “death” with frailty and risk of negative event, we found that frail people were 564% more likely to die than non-frail, and 397% more likely to die than pre-frail ones; similarly, high-risk patients were 804% more likely to die than low-risk ones, and 546% more than moderate-risk ones: as such, both SHARE-FI and MPI have proven to be reliable methods to predict 1-, 3-, and 6-month exitus. Conversely, they were not useful to predict rehospitalization in an orthogeriatric setting, according to our results. The 6-month rehospitalization rate was 11.84%, and the death rate was 13.87%. Both data are consistent with the literature, although most data referred to 1-year and not 6-month follow-up [59,60,61]. Further analysis will clarify such point in our sample. Moreover, in both cases, medical conditions rather than surgical complications were more common.

Taking co-variates into consideration, the designed regression curve revealed that higher age (OR = 1.12) and higher creatinine levels (OR = 2.66) upon admission were significantly related to death, which leads physicians to pay attention not only on age, as commonly performed, but to kidney function as well, in order to manage fluid balance and avoid exacerbations. Another regression curve demonstrated that lower folate levels (OR = 0.75) upon the admission were significantly associated with rehospitalization, and were probably tied to the significant importance that the vitamin has in several metabolic pathways, together with the relative rapidity of consumption of reserves, with respect to equally valuable molecules, such as vitamin B12 [62]. As a matter of fact, vitamin B12 levels were associated with both deaths and rehospitalizations, but with unsignificant ORs, from a clinical point of view, as well as iron levels for the sole rehospitalization. Also, it is interesting to underline that both creatinine and folate levels are related to nutritional status and quality of diet [62,63], and the fact our population was averagely at risk of malnutrition may have contributed to this matter.

Finally, by comparing autonomies in activities of daily living before the fracture and after surgery, we found a significant linear 1-month reduction, with a progressive 3- and 6-month recovery both in basic and instrumental activities, though without reaching the previous performances, meaning that, consistent with the literature on the topic [64,65,66,67], 6 months is not long enough to re-establish the *status quo ante*.

It would be useful to evaluate at least 12-month outcomes, by continuing the present study, in order to establish if the abovementioned results are also valid for a longer follow-up time.

Future studies are recommended, and should also include mobility status and risk of falling in the holistic assessment, since they are associated with increased mortality [68], and bone density evaluation [69], a crucial determinant of the pathophysiology of skeletal frailty and subsequent frailty fractures. Also, since our study did not consider AO classification, further studies may deepen the relationship with different types of fracture.

## 5. Conclusions

In conclusion, our study demonstrates that SHARE-FI and MPI are useful tools in predicting low- and mid-term deaths in orthogeriatric services. Moreover, among hematochemical exams, creatinine and folate levels are to be taken into account in the abovementioned management, given their independent association with adverse events. Finally, despite optimal medical and surgical treatment, elderly patients are unlikely to recover the lost autonomies later 6 months after surgery.

## Figures and Tables

**Table 1:** Characteristics of the sample (CGA).

Variable	Minimum	Maximum	Mean	SD
Age	65	105	83.7	7.7
SPMSQ	0	10	2.6	2.6
GDS	0	13	3.9	2.8
BADL	0	6	4.3	1.9
IADL	0	8	4.4	3.0
MNA	0	30	22.8	3.9
ESS	0	20	14.7	4.1
NPRS	0	10	1.4	2.2
CIRS Tot.	18	36	25.6	4.1
CIRS CCI	1	10	4.7	1.9
CIRS CSI	1.29	2.57	1.8	0.3
CIRS MI	2	4	3.1	0.3
MPI	0	0.813	0.3	0.2
SHARE-FI	−0.87	6.41	1.9	1.8
N. of drugs taken	0	15	4.3	2.8
N. of comorbidities	0	11	4.9	2.4
Length of hospitalization (days)	0	62	15.7	7.8
Length of stay before surgery (days)	0	37	6.2	4.7

SD, standard deviation; SPMSQ, Short Portable Mental State Questionnaire; GDS, Geriatric Depression Scale; BADL, Activities of Daily Living; IADL, Instrumental Activities of Daily Living; MNA, Mini Nutritional Assessment; ESS, Exton-Smith Scale; NPRS, Numeric Pain Rating Scale; CIRS Tot, Cumulative Illness Rating Scale; CIRS CCI, CIRS Complex Comorbidity Index; CIRS CSI, CIRS Comorbidity Severity Index; CIRS MI, CIRS Maximum Impairment; MPI, Multidimensional Prognostic Index; SHARE-FI, Survey of Health, Ageing and Retirement in Europe—Frailty Instrument; N., number.

**Table 2:** Characteristics of the sample (hematochemical exams).

Variable	Minimum	Maximum	Mean	SD
RBC (×10^6^/μL)	2.29	10.07	4.3	0.8
WBC (×10^3^/μL)	3.74	40.07	11.3	4.2
PLT (×10^3^/μL)	67	511	227.8	76.9
Hb (g/dL)	5.1	17.3	11.8	2.0
MCV (fL)	49	115	84.3	12.2
PT (INR)	0.82	3.9	1.0	0.2
aPTT (s)	17	52	29.8	4.7
Fibrinogen (mg/dL)	24	856	383.6	119.5
Glucose (mg/dL)	65	478	143	64
Creatinine (mg/dL)	0.4	4.9	1.1	0.5
Iron (μg/dL)	5	151	33.2	20.1
Transferrin (mg/dL)	97	345	181.2	38.4
Ferritin (μg/L)	9.6	1650	201.4	211.0
Total serum proteins (g/dL)	3.1	7.7	5.8	0.6
Albumin (g/dL)	1.7	4	2.9	0.4
Vitamin B12 (ng/mL)	11.5	1696	374.7	200.1
Folate (ng/mL)	1.1	26.4	7.8	5.1
TSH (μU/L)	0.024	143.701	3.1	10.1
fT4 (ng/dL)	0.52	2.35	1.4	0.3
Vitamin D (ng/mL)	4	106	18.6	14.5
PTH (pg/mL)	0.748	440.4	85.7	60.1

SD, Standard Deviation; RBC, Red Blood Cells; WBC, White Blood Cells; PLT, platelets; Hb, hemoglobin; MCV, Mean Corpuscular Volume; PT, Prothrombin Time; INR, International Normalized Ratio; aPTT, activated Partial Thromboplastin Time; TSH, Thyroid-Stimulating Hormone; fT4, free Thyroxine; PTH, Parathormone.

**Table 3:** Characteristics of the sample (comorbidities and drugs taken).

Comorbidity	Percentage
Hypertension	70.6
Heart failure	4.5
Coronary heart disease	5
Cerebrovascular disease	4.5
Atrial fibrillation	14.3
Dyslipidemia	29
History of fractures	29
Anemia	66.2
Osteoporosis	15.9
	
**Drug taken**	
Loop diuretic	18
Thiazide diuretic	18.8
Calcium channel-blocker	18.4
ACE inhibitor	27.8
Sartan	24.1
Beta-blocker	30.2
Statin	31.4
SSRI	11
SNRI	4.9
Statin	31.4
Proton pump inhibitor	26.1
Benzodiazepine	23.7
Opioid	3.7

ACE, angiotensin-converting enzyme; SSRI, selective serotonin reuptake inhibitors; SNRI, serotonin–norepinephrine reuptake inhibitors; anemia, hemoglobin < 13 g/dL [45].

**Table 4:** Hazard ratios (weighted for MPI and SHARE-FI) with 95% C.I.

MPI	Low Risk	Moderate Risk	High Risk		MPI	Low Risk	Moderate Risk	High Risk
Low risk	-	**5.46**	**8.04**		Low risk	-	1.69	4.08
		**2.59 to 11.48**	**1.97 to 32.75**				0.76 to 3.76	0.85 to 19.55
Moderate risk	**0.18**	-	1.47		Moderate risk	0.59	-	2.41
	**0.08 to 0.38**		0.34 to 6.37			0.26 to 1.31		0.47 to 12.31
High risk	**0.12**	0.68	-		High risk	0.24	0.41	-
	**0.03 to 0.51**	0.16 to 2.93				0.05 to 1.17	0.08 to 2.13	
								
SHARE-FI	Non-frail	Pre-frail	Frail		SHARE-FI	Non-frail	Pre-frail	Frail
Non-frail	-	1.42	**5.64**		Non-frail	-	0.47	1.74
		0.42 to 4.80	**2.00 to 15.91**				0.15 to 1.48	0.65 to 4.68
Pre-frail	0.70	-	**3.97**		Pre-frail	2.12	-	3.69
	0.21 to 2.38		**1.25 to 12.59**			0.68 to 6.63		1.24 to 11.03
Frail	**0.18**	**0.25**	-		Frail	0.57	0.27	-
	**0.06 to 0.49**	**0.08 to 0.79**				0.21 to 1.54	0.09 to 0.81	
		
EXITUS					REHOSPITALIZATION			

MPI, Multidimensional Prognostic Index; SHARE-FI, Survey of Health, Ageing and Retirement in Europe—Frailty Instrument.

**Table 5:** Cox regressions (y = exitus).

Variable	Standard Error	OR	95% C.I.	*p*
Age	0.0581	1.12	1.002–1.258	**0.046**
BADL	0.1632	0.73	0.534–1.012	0.059
Creatinine	0.3344	2.66	1.381–5.123	**0.003**
Vitamin B12	0.0004	1.004	1.002–1.006	**<0.0001**

OR, odds ratio; C.I., confidence interval; BADL, Activities of Daily Living.

**Table 6:** Cox regressions (y = rehospitalization).

Variable	Standard Error	OR	95% C.I.	*p*
Iron	0.012	1.03	1.003–1.052	**0.026**
Vitamin B12	0.001	1.002	1.0003–1.004	**0.025**
Folate	0.130	0.75	0.584–0.973	**0.03**

OR, odds ratio; C.I., confidence interval.

## Data Availability

The data and materials used and/or analyzed during the current study are not publicly available. They are available from the corresponding author upon reasonable request.

## Abbreviations

ACE, angiotensin-converting enzyme; aPTT, activated partial thromboplastin time; BADL, Activities of Daily Living; C.I., confidence interval; CIRS Tot, Cumulative Illness Rating Scale; CIRS CCI, CIRS Complex Comorbidity Index; CIRS CSI, CIRS Comorbidity Severity Index; CIRS MI, CIRS Maximum Impairment; CGA, Comprehensive Geriatric Assessment; ESS, Exton-Smith Scale; fT4, free thyroxine; GDS, Geriatric Depression Scale; Hb, hemoglobin; HR, hazard ratio; IADL, Instrumental Activities of Daily Living INR, international normalized ratio; MCV, mean corpuscular volume; MNA, Mini Nutritional Assessment; MPI, Multidimensional Prognostic Index; N., number; NPRS, Numeric Pain Rating Scale; OR, odds ratio; PLT, platelets; PT, prothrombin time; PTH, parathormone; RBCs, red blood cells; SD, standard deviation; SHARE-FI, Survey of Health, Ageing and Retirement in Europe—Frailty Instrument; SNRI, serotonin –norepinephrine reuptake inhibitors; SPMSQ, Short Portable Mental State Questionnaire; SSRIs, selective serotonin reuptake inhibitors; TSH, thyroid-stimulating hormone; WBCs, white blood cells.
